# Effective Management of Severe Epidermal Growth Factor Receptor Inhibitors-Induced Papulopustular Eruption With Low-Dose Isotretinoin and Corticosteroids

**DOI:** 10.7759/cureus.72878

**Published:** 2024-11-02

**Authors:** Beatrice Bălăceanu-Gurău, Andra Copilau, Alexandra Timofte, Cristian-Dorin Gurau, Mara Madalina Mihai

**Affiliations:** 1 Oncologic Dermatology, Carol Davila University of Medicine and Pharmacy, Bucharest, ROU; 2 Dermatology, Elias Emergency University Hospital, Bucharest, ROU; 3 Orthopedics and Traumatology, Carol Davila University of Medicine and Pharmacy, Bucharest, ROU; 4 Orthopedics and Traumatology, Clinical Emergency Hospital, Bucharest, ROU; 5 Botany and Microbiology, Research Institute of the University of Bucharest, Bucharest, ROU

**Keywords:** acneiform rash, cutaneous adverse effects, epidermal growth factor receptor inhibitors, isotretinoin, panitumumab, systemic retinoids

## Abstract

Epidermal growth factor receptor inhibitors (EGFRi) are approved for treating various cancers. Given that EGFR signaling is crucial for normal skin growth and repair, inhibiting this pathway can disrupt skin homeostasis and integrity. Although generally well tolerated, molecularly targeted therapies can lead to skin-related adverse effects that significantly impact patients’ quality of life, often resulting in treatment interruptions. The most common cutaneous reaction associated with EGFRi is a diffuse, papulopustular acneiform eruption. Management strategies can be tailored based on the severity of the condition and may include topical corticosteroids, topical and oral antibiotics, and systemic corticosteroid therapy. Moreover, consistent evidence supports the effectiveness of systemic retinoids at lower doses than those typically prescribed for acne vulgaris, advocating for their use when other treatments have failed. Herein, we present the case of a 71-year-old male with advanced colorectal cancer who was treated with panitumumab and subsequently developed a generalized papulopustular eruption three weeks after initiating therapy. The patient reported pruritus, a burning sensation, and hyperesthesia, which significantly impacted his daily activities. Physical examination revealed folliculocentric erythematous pustules and papules covering more than 30% of his body surface area (including the face, scalp, thorax, and extremities), along with xerosis, painful nasal and oral mucosal erosions, paronychia, and trichomegaly. Initial treatment with topical and systemic antibiotics, topical and systemic corticosteroids, and general skin care yielded poor results, with recurring moderate to severe eruptions occurring every 7 to 10 days. After three months and with approval from the oncology team, isotretinoin therapy (10 mg/day) was initiated under close monitoring of laboratory parameters. This approach achieved effective therapeutic control within one month, allowing the continuation of oncologic treatment without necessitating any dose modifications. In such cases, dermatologists play a vital role in managing these adverse reactions, ensuring that effective treatments enable patients to continue lifesaving oncologic therapies.

## Introduction

Cancers originating from epithelial cells are characterized by mutations affecting growth factors and their receptors, resulting in uncontrolled cellular proliferation, migration, and angiogenesis [1-3]. In this context, epidermal growth factor receptor inhibitors (EGFRi) disrupt this signaling pathway, providing a treatment option for various epithelial malignancies [1,3,4]. Currently, EGFRi are approved for managing non-small-cell lung cancer, head and neck squamous-cell carcinoma, pancreatic cancer, and colorectal cancer, as well as malignancies of the large bowel, stomach, ovary, and breast [1,4].

EGFRi can be classified based on various criteria, including chemical structure, reversibility, class, and pharmacological effects. In oncologic therapies, they fall into two categories: tyrosine kinase inhibitors (TKIs) and monoclonal antibodies [1,4]. TKIs inhibit EGFR signaling by binding to the receptor’s intracellular catalytic domain, preventing phosphorylation and thereby obstructing downstream signaling [1,4]. In contrast, humanized monoclonal antibodies target the extracellular domain of EGFR, competitively inhibiting ligand binding [1,4].

Physiologically, EGFR is predominantly expressed in the epidermis, particularly in the basal layer [1,5]. This signaling pathway regulates cutaneous homeostasis through mechanisms that promote pro-survival and antiapoptotic effects [5]. The primary physiological function of EGFR is to regulate the development and stability of epithelial tissues [5]. Chronic pharmacological inhibition of EGFR can lead to localized keratinocyte necrosis, resulting in sustained recruitment and activation of immune cells [1-5].

Given that EGFR is present in multiple solid tumors as well as in basal and suprabasal keratinocytes, skin appendages (such as sebaceous epithelium and eccrine glands), the outer root sheath of hair follicles, and dermal capillary endothelium, dermatologic issues are frequently observed in patients undergoing treatment with EGFRi [1,5-8]. Inhibiting EGFR compromises skin integrity and homeostasis, weakening the outermost layer and ultimately resulting in dryness and fissure formation [4,5]. Additionally, it alters sebum production and impairs the skin’s protective function, increasing permeability and susceptibility to injury, irritation, and bacterial infections [1,5]. The release of pro-inflammatory cytokines in the skin and the activation of inflammatory cells further heighten skin vulnerability during EGFRi treatment [9,10]. Consequently, cutaneous complications are the most frequent adverse side effects associated with EGFRi.

While the precise mechanisms underlying EGFRi-induced cutaneous changes remain unclear, it is believed that cutaneous abnormalities arise from alterations in EGFR signaling that affect cell growth and provoke inflammation around hair follicles, along with genetic changes linked to activation signals [1,8]. Notably, these inhibitors interfere with the RAS/RAF/MEK/ERK pathway, impacting cell cycle regulation, including the proliferation and differentiation of epidermal cells [8].

This article was previously presented as a poster presentation at the 2024 European Academy of Dermatology and Venereology Congress on September 25-28, 2024.

## Case presentation

We present the case of a 71-year-old male with advanced colorectal cancer involving the upper bladder wall, several loco-regional lymph nodes, and multiple liver metastases. He underwent treatment with panitumumab, a humanized monoclonal antibody targeting EGFR. Three weeks after initiating therapy, the patient developed a generalized acneiform rash. He experienced pruritus, a burning sensation, and hyperesthesia, which significantly affected his daily activities.

**Figure 1:**
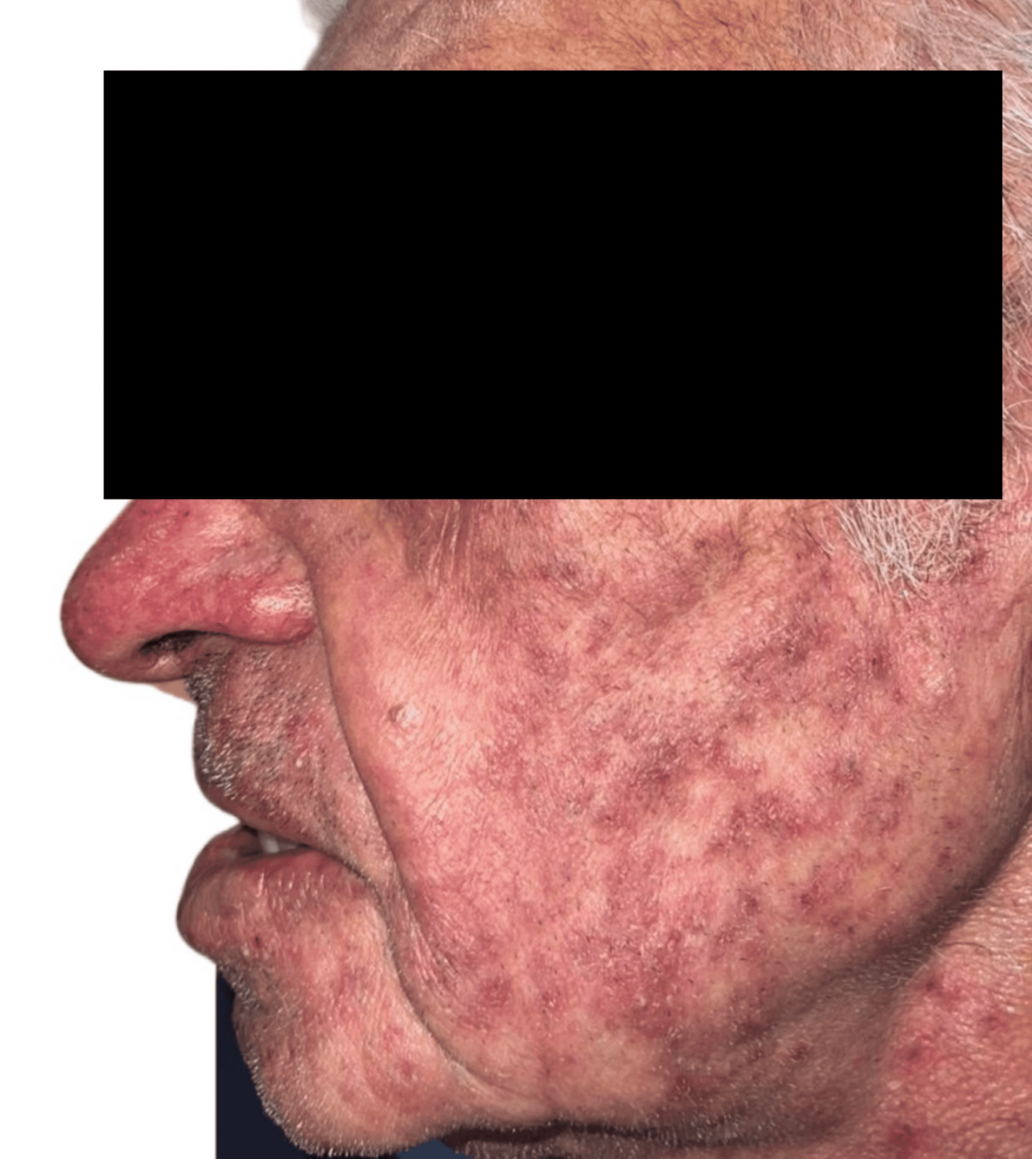
Diffuse acneiform rash observed on the left cheek Folliculocentric erythematous pustules and papules, characterized by the absence of comedones or purulent cysts.

**Figure 2:**
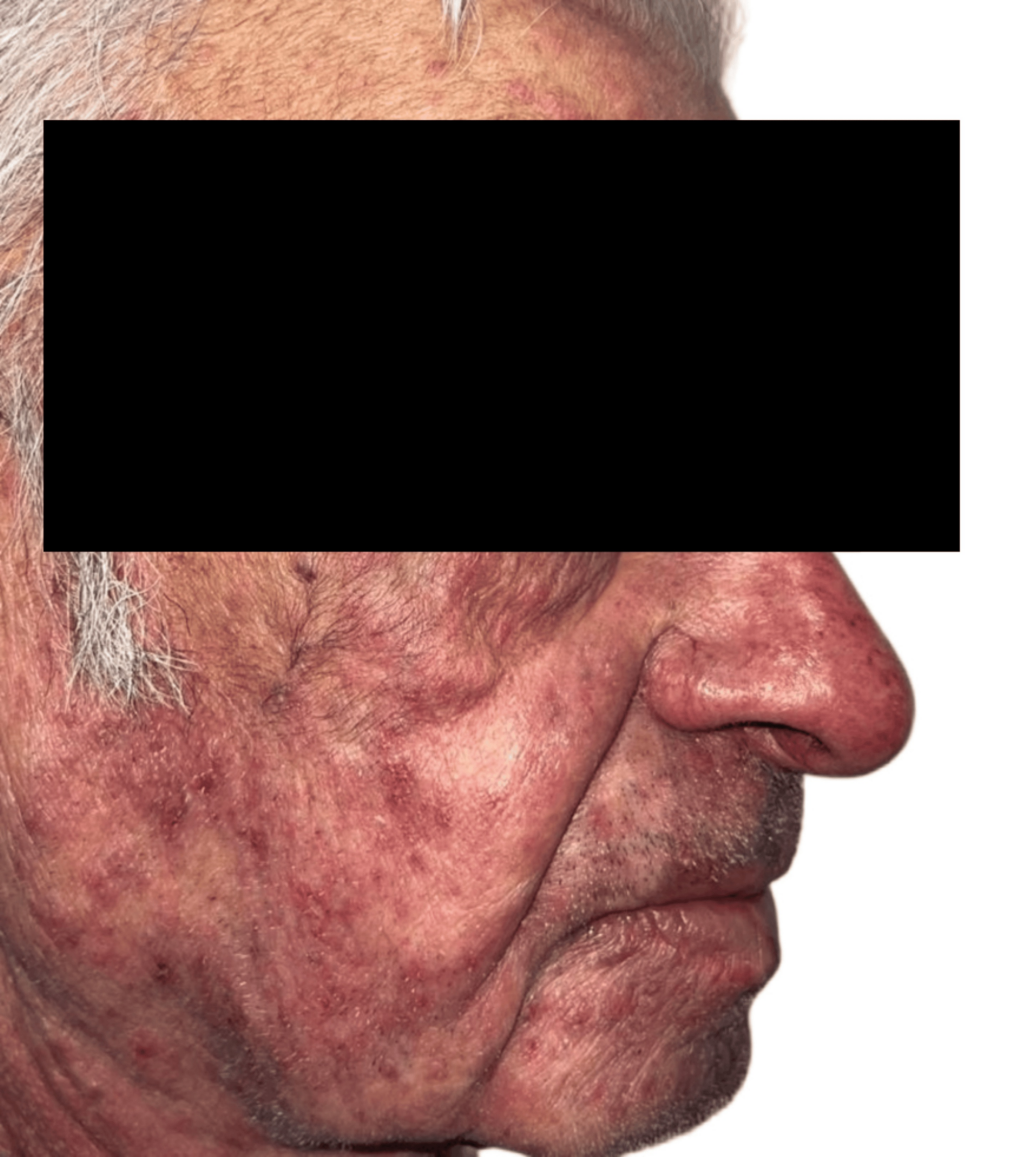
Diffuse acneiform rash observed on the right cheek Folliculocentric erythematous pustules and papules, characterized by the absence of comedones or purulent cysts.

**Figure 3:**
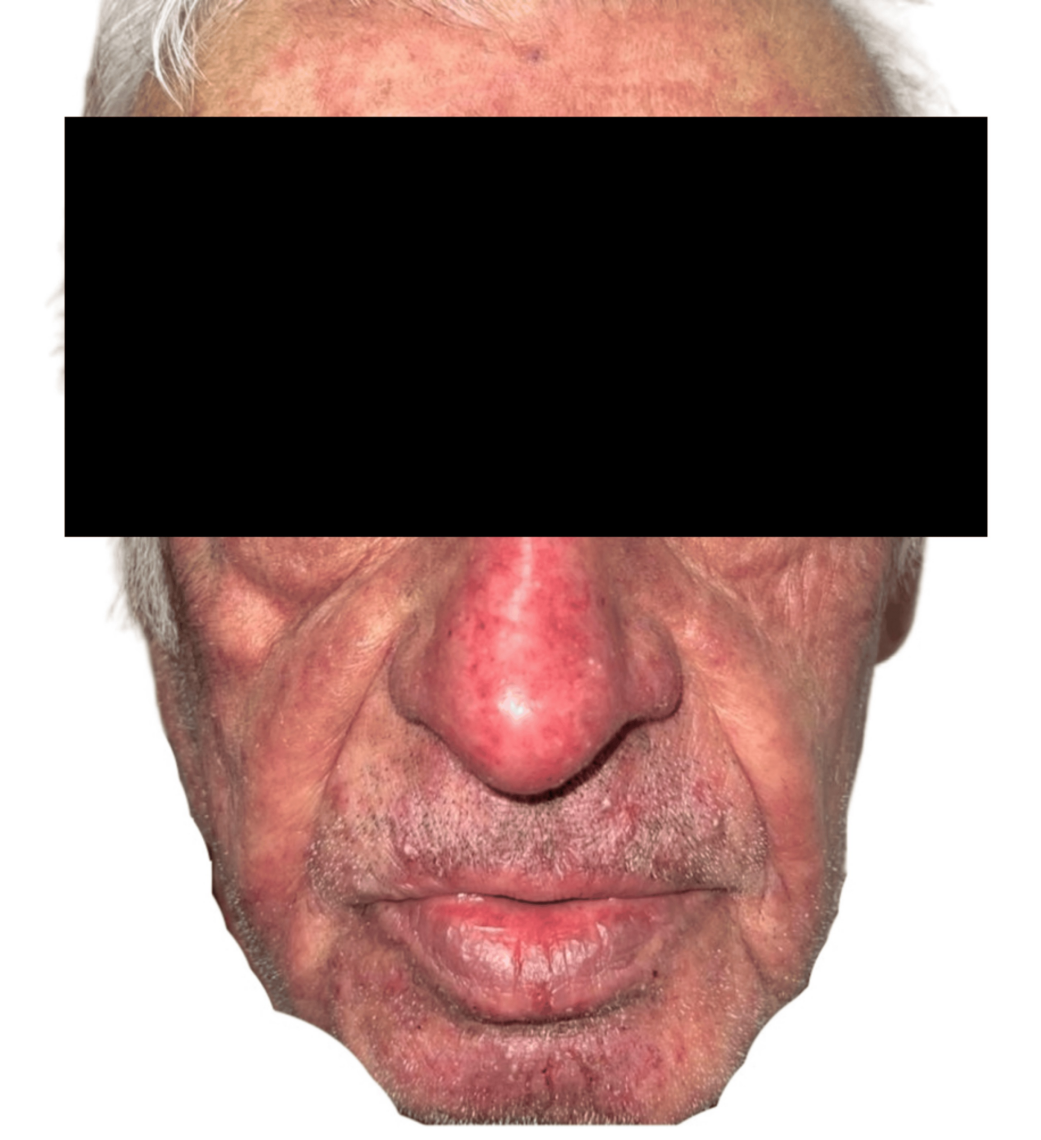
Diffuse acneiform rash observed on the face Folliculocentric erythematous pustules and papules, characterized by the absence of comedones or purulent cysts.

**Figure 4:**
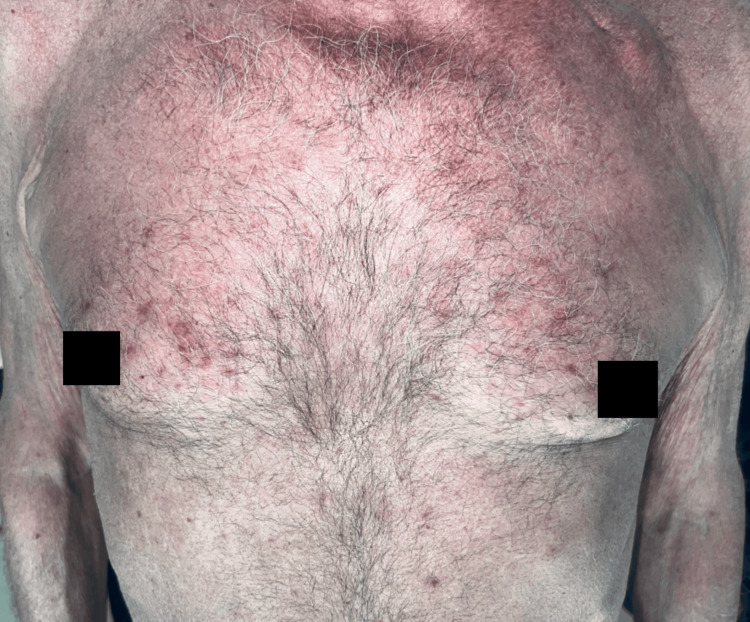
Acneiform rash on the anterior chest, accompanied by xerosis

**Figure 5:**
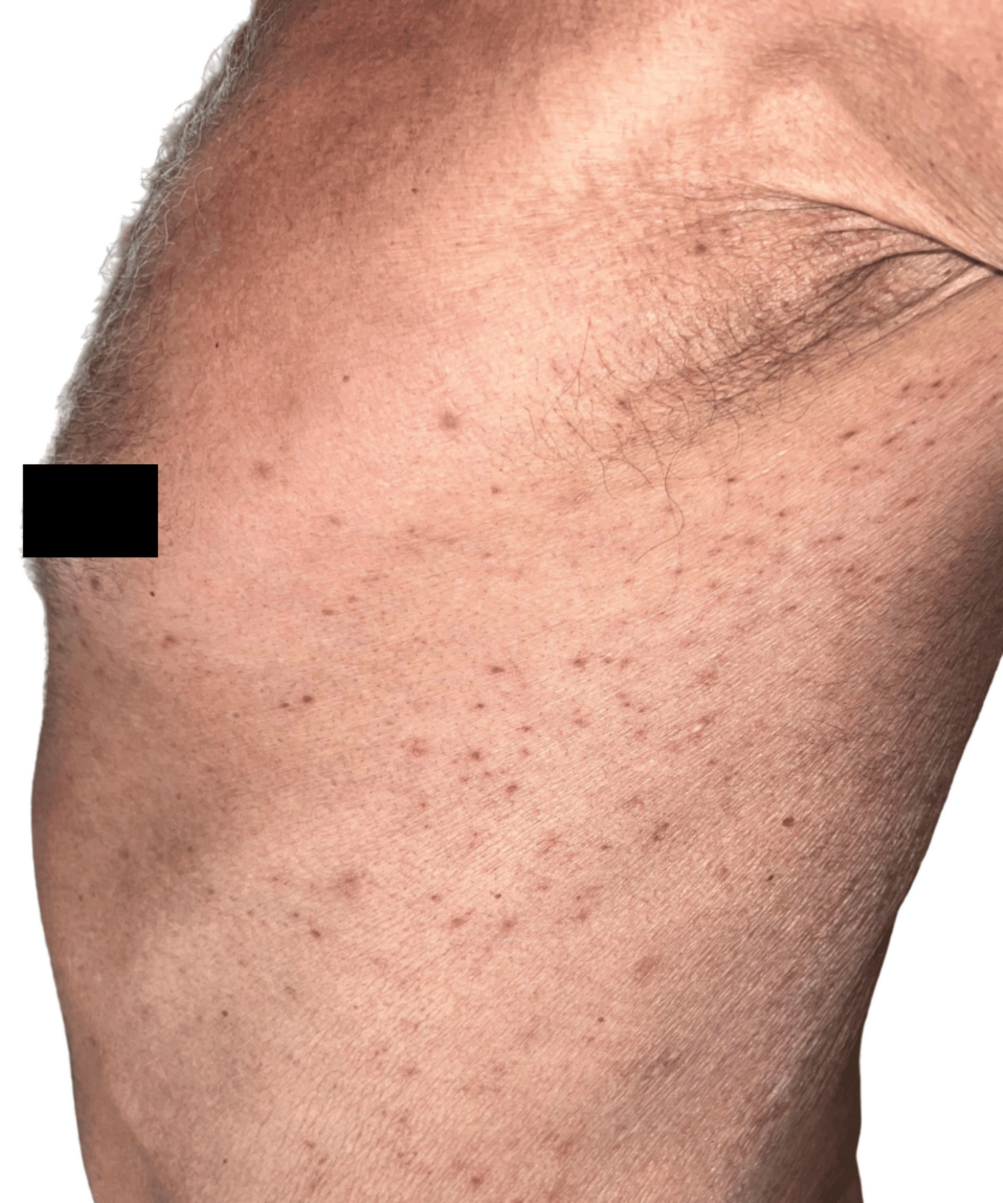
Papules and pustules located on the truck

**Figure 6:**
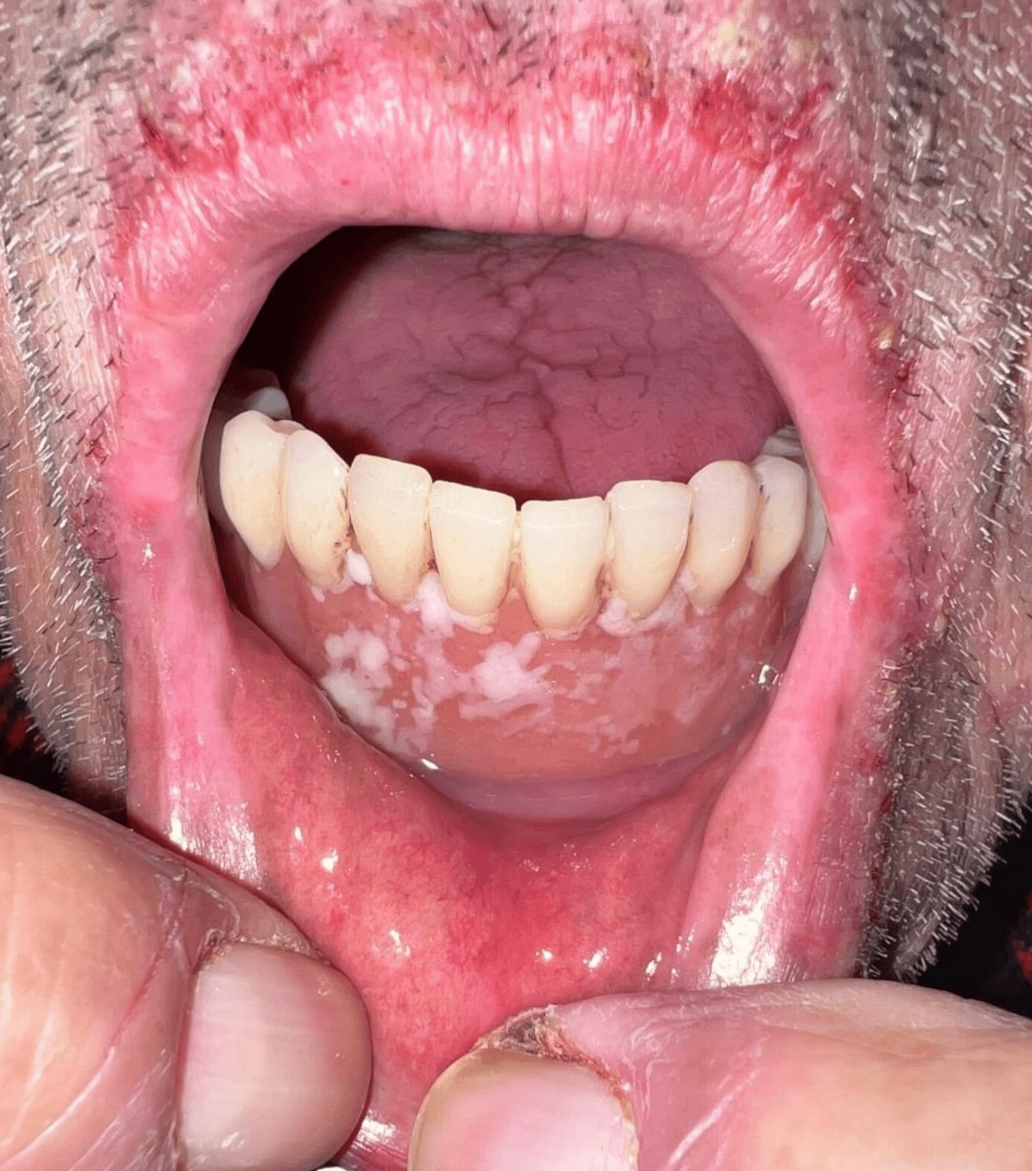
Oral mucositis

**Figure 7:**
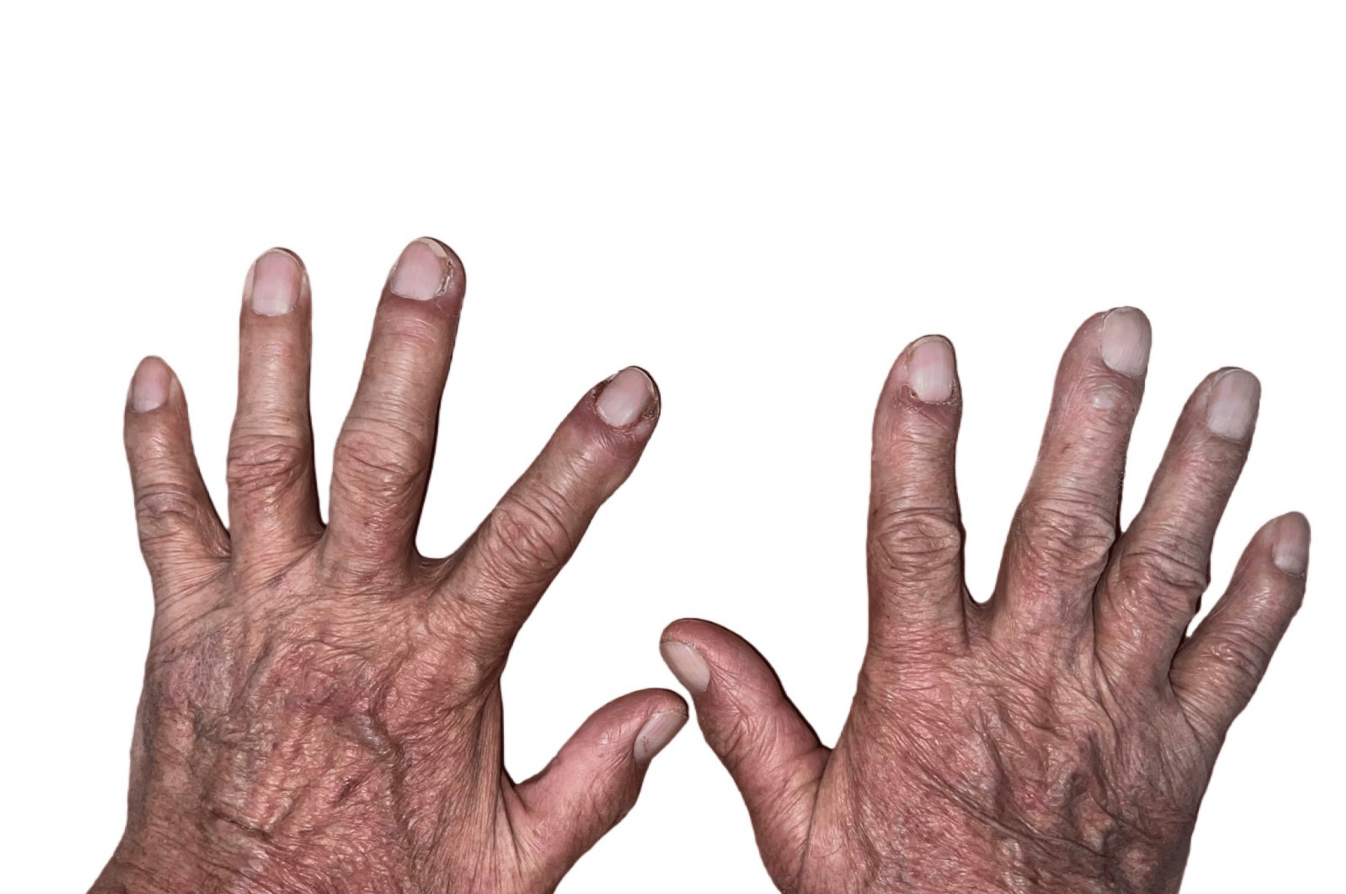
Paronychia of the second and third fingers of the left hand

Physical examination revealed inflammatory folliculocentric erythematous pustules and papules covering over 30% of the body surface area (BSA), including the face, scalp, anterior chest, and extremities (Figure 1, Figure 2, Figure 3, Figure 4, Figure 5). Other findings included generalized xerosis (Figure 4, Figure 5), erythema, and painful erosions of the nasal and oral mucosa (Figure 6), as well as paronychia (Figure 7) and trichomegaly. Laboratory tests indicated a mild inflammatory biological syndrome, with the erythrocyte sedimentation rate measured at 33 mm/h (normal range: 0-15 mm/h) and the CRP level recorded at 18 mg/dL (normal range: <0.5 mg/dL).

The patient was diagnosed with a severe acneiform rash induced by EGFRi treatment, characterized by papules and pustules affecting more than 30% of the BSA and resulting in severe symptoms that significantly limited self-care activities of daily living (ADLs).

He was prescribed low-potency topical corticosteroids (hydrocortisone 1%) to be applied twice daily to the affected areas in conjunction with oral doxycycline (100 mg twice daily) for four weeks. Topical high-potency glucocorticoids were administered for nail involvement, and boro-glycerine was prescribed to manage mucosal lesions. General care measures included avoiding frequent washing with hot water and harsh soaps, detergents, solvents, or disinfectants; minimizing sun exposure; using a broad-spectrum sunscreen; and applying emollients twice daily.

Upon reevaluation after four weeks, no improvement was noted in the cutaneous eruption. Bacteriologic swabs taken from local pustules revealed a superinfection with *Staphylococcus aureus*, prompting the prescription of a first-generation oral cephalosporin, cephalexin (500 mg twice daily) for four weeks, along with topical treatment using fusidic acid.

Given the lack of clinical improvement at the follow-up visit four weeks later, oral prednisone at a dosage of 5 mg/day was initiated for one month. After three months of treatment with the aforementioned therapeutic agents, the patient exhibited a poor therapeutic response, experiencing recurrences of moderate to severe eruptions every seven to 10 days following each administration of panitumumab. In light of these challenges, and under the oncologist’s supervision with close monitoring of laboratory parameters (liver function tests and lipid levels) and patient symptoms, low-dose systemic isotretinoin (10 mg/day) was initiated.

**Figure 8:**
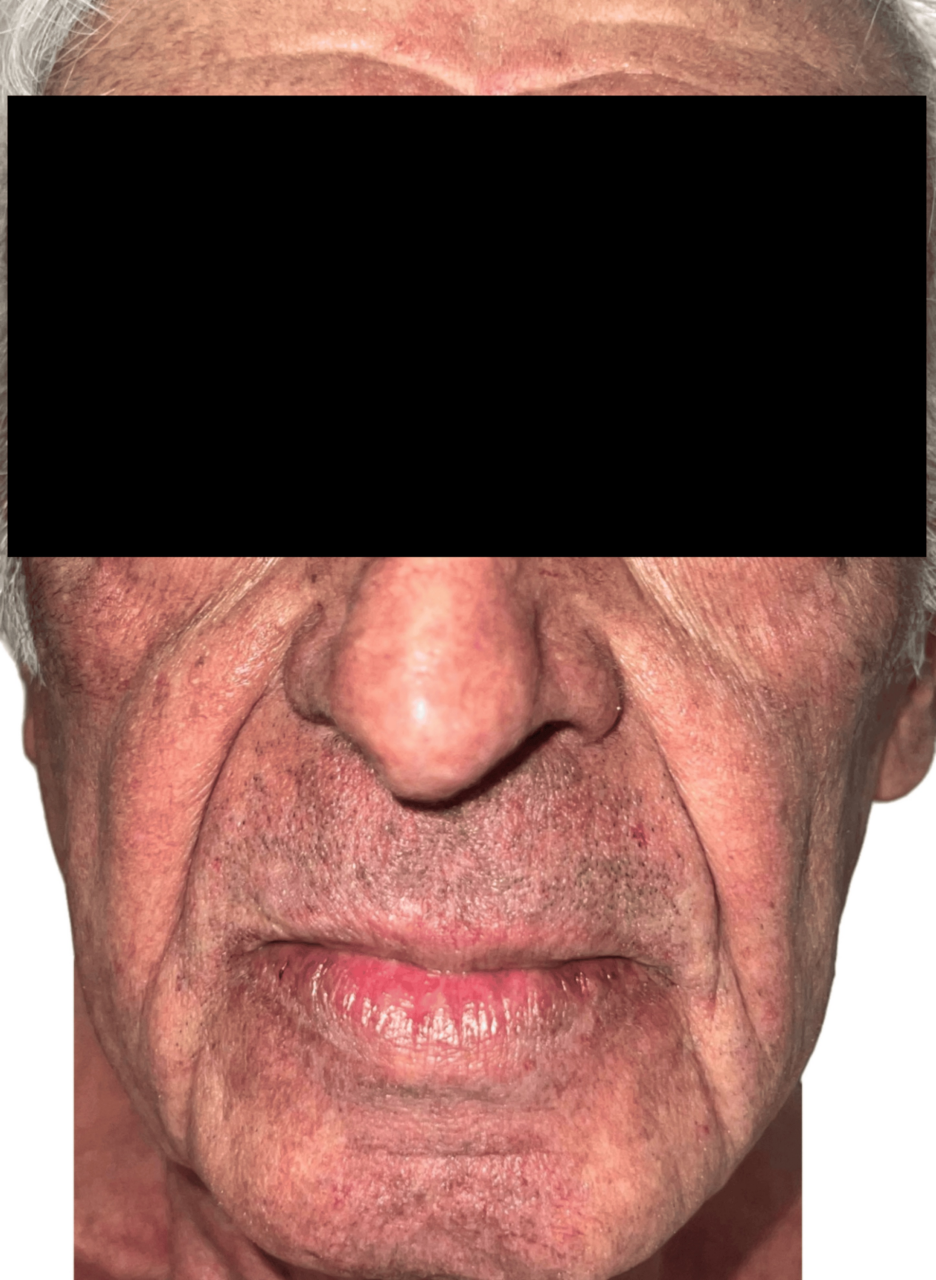
Clinical improvement of facial lesions after one month of low-dose oral isotretinoin therapy

**Figure 9:**
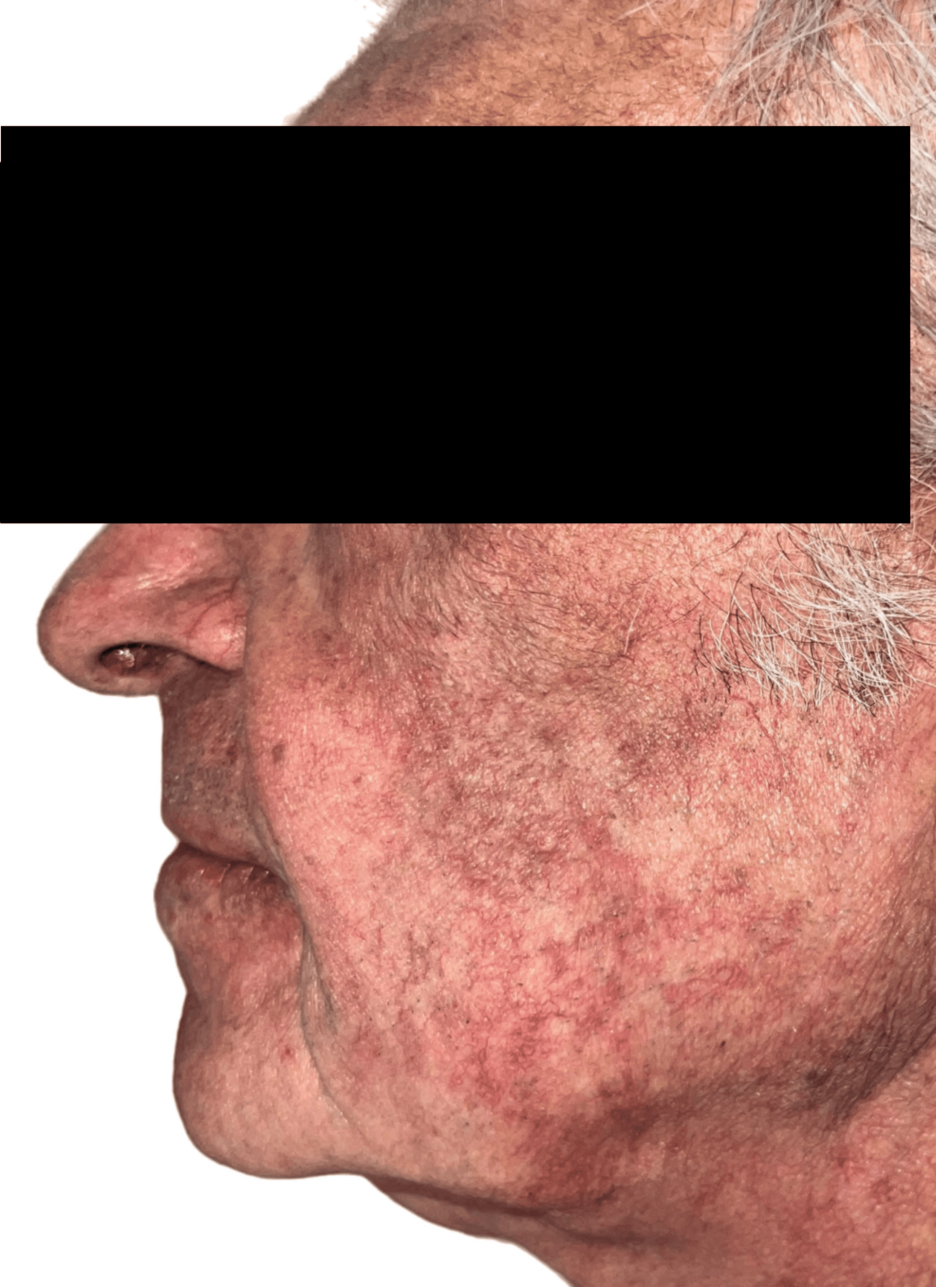
Clinical improvement of facial lesions after one month of low-dose oral isotretinoin therapy

After one month of therapy with low-dose systemic isotretinoin (10 mg/day) and low-dose systemic prednisone (5 mg/day), the patient showed significant clinical improvement of the acneiform rash, enabling the continuation of oncologic therapy without the need for dose adjustments (Figure 8, Figure 9). No significant alterations in liver function tests, renal function tests, or lipid levels were detected at one-month and three-month follow-up assessments, and there was no need for additional dermatologic treatment. Given that isotretinoin may induce or exacerbate xerosis, photosensitivity, and cheilitis, the use of emollients and photoprotection was maintained throughout the treatment.

## Discussion

EGFRi can induce a range of skin reactions, including acneiform eruptions, xerosis, pruritus, paronychia, changes in hair, mucous membrane alterations, and, rarely, severe cutaneous reactions such as Stevens-Johnson and toxic epidermal necrolysis [1,4]. Initial cutaneous manifestations typically arise within the first two weeks of treatment in over 75% of patients, although they may also develop up to two months after treatment initiation [1,8,9]. Erythema and edema are usually the first signs, often accompanied by pain, itching, irritation, and a burning sensation [1,8,9]. Subsequently, during the second to fourth weeks, patients may experience folliculitis and/or pustular lesions, which may be associated with pruritus [1,9,10]. In later stages, complications such as purpura and ulcers may occur, particularly on the buttocks and lower extremities [1,8,9]. These skin-related complications can pose a risk of interrupting, reducing, or discontinuing cancer therapies. Therefore, understanding how to identify and effectively manage these side effects is essential.

Severe skin reactions associated with EGFRi can be categorized by various risk factors, including treatment specifics, tumor characteristics, patient demographics, and environmental influences [1,2]. The specific EGFRi used significantly impacts the frequency and severity of skin eruptions [10,11]. For instance, panitumumab and cetuximab are associated with higher reaction rates compared to other monoclonal antibodies and TKIs [10,11]. Increasing the dosage or resuming treatment can further exacerbate skin reactions [10,11]. Additionally, skin regions that have previously undergone radiation exposure may remain unaffected by eruptions during EGFRi therapy; however, an increased incidence of skin reactions has been observed in patients receiving combined therapy with EGFRi and radiation [12]. Patients with colorectal cancer exhibit a higher incidence of acneiform eruptions, potentially due to unique tumor biology that influences skin susceptibility to drug toxicity [13].

Patient-related factors also play a significant role in the occurrence of skin reactions. Individuals with fair skin may experience more severe eruptions, although some studies have found no correlation [1,14,15]. Younger individuals and males are at higher risk, while older patients may experience reduced cutaneous symptoms due to lower EGFR expression [14,15]. Enhanced immune performance may correlate with more pronounced skin reactions; conversely, EGFRi can reduce the skin’s antimicrobial defenses and disrupt the skin barrier, increasing susceptibility to infections, particularly with Staphylococcus aureus [5,16]. Clinical signs include increased erythema, swelling, and pus formation, with management typically involving oral antibiotics and topical treatments to reduce inflammation [5,16]. Regular monitoring and patient education on signs of superinfection are essential for effective management [5,16].

Interestingly, a papulopustular rash induced by EGFRi treatment is associated with improved overall survival and progression-free survival in patients receiving anti-EGFR monoclonal antibodies and TKIs [13]. Consequently, the presence of these skin eruptions can serve as a prognostic indicator of tumor response to therapy [13].

Cutaneous side effects can be categorized using various scales, including the National Cancer Institute Common Terminology Criteria for Adverse Events (CTCAE) system 5.0 (Table 1) [17].

**Table 1:** CTCAE severity grading of acneiform rashes induced by EGFRi ADL, activities of daily living; BSA, body surface area; CTCAE, Common Terminology Criteria for Adverse Events; EGFRi, epidermal growth factor receptor inhibitors

Grades	Clinical manifestations
Grade 1 (mild)	Papules and/or pustules affecting <10% of the BSA, associated or not with symptoms of pruritus or tenderness.
Grade 2 (moderate)	Papules and/or pustules affecting 10-30% of the BSA, associated or not with symptoms of pruritus or tenderness; associated with psychosocial impact; limiting instrumental ADL; papules and/or pustules affecting >30% of the BSA, with or without mild symptoms.
Grade 3 (severe)	Papules and/or pustules affecting >30% of the BSA, with moderate or severe symptoms; limiting self-care ADL; associated with local superinfection with oral antibiotics indicated.
Grade 4 (life-threatening)	Life-threatening consequences; papules and/or pustules covering any percent of the BSA, associated or not with symptoms of pruritus or tenderness, and are associated with extensive superinfection with intravenous antibiotics indicated.
Grade 5 (fatal)	Death

Patients undergoing EGFRi therapy experience increased sensitivity to irritants and allergens, necessitating careful skincare practices to prevent exacerbation [1,5]. General recommendations for managing skin reactions from EGFRi include avoiding frequent washing with hot water, using harsh soaps, detergents, solvents, or disinfectants, and limiting sun exposure [1,5]. It is advisable to apply a broad-spectrum mineral sunscreen every two hours and use emollients twice daily [5]. Given the heightened incidence of cutaneous eruptions during the initial two to four weeks of EGFRi therapy, proactive management is recommended unless contraindicated by patient or healthcare professional considerations.

The management of acneiform eruptions varies based on severity. For a Grade 1 rash, topical low-potency corticosteroids (such as hydrocortisone 1%) and antibiotics (such as clindamycin or erythromycin) are recommended [5,17]. For a Grade 2 rash, oral doxycycline (100 mg twice daily for four to six weeks) along with topical low-potency corticosteroids is suggested [5,17]. Alternatively, minocycline (100 mg once or twice daily) has proven effective in reducing lesion numbers within the first eight weeks, although doxycycline is preferred due to its superior safety profile, especially for patients with renal dysfunction [5]. If the patient is already receiving prophylactic tetracycline, alternatives such as first-generation oral cephalosporins (e.g., cephalexin or cefadroxil) or trimethoprim-sulfamethoxazole can be considered for four weeks [5]. When bacterial superinfection is suspected, cultures should be performed when feasible. For a Grade 3 rash, treatment adjustments - including dose changes or interruptions of oncologic treatment - should be made in consultation with both the oncologist and the patient [5,17]. Medium- to high-potency topical corticosteroids may also be recommended, supported by studies demonstrating inflammatory chemokine release following EGFRi therapy [5].

Isotretinoin, a retinoid, functions by reducing sebaceous gland activity, decreasing keratinocyte proliferation, exhibiting anti-inflammatory properties, and influencing gene expression related to skin cell turnover [18-20]. Growing evidence supports the effectiveness of isotretinoin in managing EGFRi-induced acneiform rashes, particularly at lower doses than those typically employed for acne vulgaris, especially when conventional treatments have proven ineffective [1,18-20]. The advantage of isotretinoin lies in its ability to diminish lesion severity and prevent treatment interruptions, thereby enabling patients to continue with potentially lifesaving oncologic therapies [18-20]. However, further studies on isotretinoin for EGFRi-induced cutaneous rashes, including case-control studies, are warranted [1,18-20].

## Conclusions

In the realm of molecularly targeted therapies, particularly EGFRi, dermatologic toxicities present a frequent and significant concern, with acneiform rashes being the most commonly reported side effect. It is crucial to recognize that papulopustular cutaneous eruptions induced by EGFRi not only signify treatment-related adverse effects but also hold prognostic importance, as their severity has been positively correlated with overall survival and progression-free survival in patients. However, these skin manifestations can adversely affect patient adherence to potentially lifesaving oncologic treatments due to discomfort, disfigurement, and disruption of daily activities.

This case report highlights the successful use of isotretinoin as an effective therapeutic strategy for managing severe EGFRi-induced acneiform rash. Despite the known risk of adverse effects, isotretinoin resulted in significant clinical improvement, allowing the patient to continue oncologic therapy without necessitating dose adjustments. This case emphasizes the potential of considering isotretinoin at lower doses as a viable option when conventional treatments are ineffective, thereby illustrating its role in mitigating cutaneous side effects associated with targeted cancer therapies. Future studies should focus on further evaluating the safety and efficacy of isotretinoin in similar contexts to establish standardized treatment protocols for managing skin toxicities in cancer patients.
